# Reduced liver cancer mortality with regular clinic follow‐up among patients with chronic hepatitis B: A nationwide cohort study

**DOI:** 10.1002/cam4.3421

**Published:** 2020-08-28

**Authors:** Jae‐Jun Shim, Gi‐Ae Kim, Chi Hyuk Oh, Jung Wook Kim, Jisun Myung, Byung‐Ho Kim, In‐Hwan Oh

**Affiliations:** ^1^ Department of Internal Medicine Kyung Hee University School of Medicine Seoul Korea; ^2^ Department of Preventive Medicine School of Medicine Kyung Hee University Seoul Korea

**Keywords:** adult liver cancer, chronic hepatitis B, mortality, office visits

## Abstract

**Background:**

Regular clinic follow‐up is a prerequisite for optimal antiviral therapy and surveillance of hepatocellular carcinoma in patients with chronic hepatitis B (CHB). However, adherence to regular follow‐up stays low in practice. This study investigated whether regular follow‐up is associated with decreased liver cancer mortality in CHB patients.

**Methods:**

A nationwide population‐based historical cohort study was conducted using customized data from the National Health Insurance Service of Korea. The number of hospital visits every 3‐month interval was counted for 2 years from the date of CHB diagnosis. Patients were classified into three follow‐up groups: regular (four to eight visits), irregular (one to three visits), and no follow‐up. The risk of liver cancer mortality was compared among the groups using Cox proportional hazard regression analysis.

**Results:**

Of the 414 074 CHB patients, 22.9% had regular follow‐up. In multivariable analysis, regular follow‐up was independently associated with decreased risk of liver cancer mortality compared to no follow‐up (hazard ratio [HR], 0.56; 95% confidence interval [CI], 0.50‐0.63, *P* < .001). Regular follow‐up was also associated with the lowest risk of all‐cause mortality (HR, 0.60; 95% CI, 0.57‐0.63, *P* < .001). Patients with regular follow‐up received more curative treatment (23.1% vs 15.1%, *P* < .001). Patients were less motivated when they were female, >60 years, of low socioeconomic status, disabled, lived in a rural area, had a higher comorbidity rate, or did not have cirrhosis.

**Conclusions:**

Regular follow‐up at least every 3‐6 months is significantly associated with reduced liver cancer mortality in patients with CHB.

## Graphical abstract

Of the 414 074 CHB patients, 22.9% had regular follow‐up. Regular follow‐up was independently associated with decreased risk of liver cancer mortality compared to no follow‐up (hazard ratio [HR], 0.56; 95% confidence interval [CI], 0.50‐0.63, *P* < .001). Regular follow‐up at least every 3‐6 months can significantly reduce liver cancer mortality in patients with CHB.

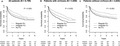

## 1 INTRODUCTION

Approximately 291 million people are chronically infected with the hepatitis B virus (HBV) worldwide and the mortality rate due to HBV‐related liver cancer is projected to double by 2040.^1^, ^2^, ^3^ With the overall prognosis of liver cancer remaining dismal, current guidelines recommend regular surveillance for hepatocellular carcinoma (HCC) and long‐term antiviral treatment in patients with chronic hepatitis B (CHB).^4^, ^5^, ^6^ To optimize these interventions, however, patients should maintain long‐term medical care.

Regular follow‐up involves periodic medical visits for the management of a chronic disease. Missing scheduled visits is not uncommon in clinical practice, which brings challenging situations to clinicians including an increased risk of hospital admission, poor cancer screening, and increased mortality.^7^, ^8^, ^9^ Caring for patients with CHB is ongoing and arduous, and the continuous participation of patients is essential for long‐term treatment. However, only a small percentage of patients with CHB undergo regular surveillance for liver cancer and receive timely antiviral treatment.^10^, ^11^, ^12^, ^13^ It has been reported that <10% of patients with cirrhosis receive consistent screening before their cancer diagnosis.^14^

Strong supporting evidence for the beneficial outcomes of regular follow‐up of CHB is lacking, with few supporting data on the beneficial effects of regular follow‐up in patients with CHB. If its effects are clearly proven, clinicians can recommend regular follow‐up more confidently and find more innovative methods to improve the adherence rate. Therefore, this study conducted a large nationwide population‐based observational study of the association between regular follow‐up and risk of liver cancer mortality in patients with CHB.

## 2 METHODS

### 2.1 Data source

A nationwide, population‐level, historical cohort study was conducted using data from the National Health Insurance Service (NHIS). NHIS achieved universal coverage of medical care in 1989 and currently involves >99% of the entire population (over 50 million) in the Republic of Korea (ROK). The service includes all health care providers in the ROK, which makes it possible to trace patient information even for patients treated at various facilities.

We uses the Korean version of the International Classification of Diseases to define CHB (B18.0 or B18.1), cirrhosis (K70.2, K70.3, K71.7, K74, K76.1, or K76.6), and liver cancer (C22.0, C22.1, or C22.9).^15^

From this database, customized and anonymized data on healthcare utilization, sociodemographic variables, and mortality were obtained.^16^ A historical cohort of adult patients older than 20 who were diagnosed with CHB between January 2009 and December 2013 was recruited from the source data. The prevalence of hepatitis B surface antigen positivity in the general population of the ROK during the study period was 3.0%.^17^, ^18^

### 2.2 Study population

**Figure 1:**
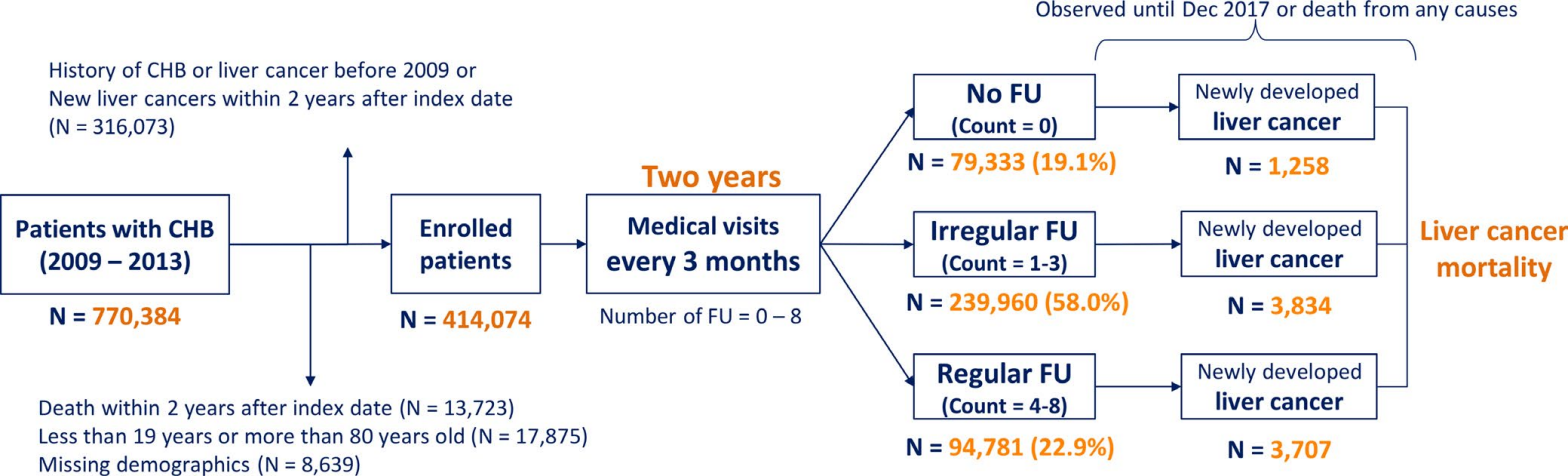
Flowchart of study population. CHB, chronic hepatitis B

This study included 770 384 adult patients with CHB who had visited outpatient clinics at least twice or had been admitted at least once with a disease code of CHB from January 2009 to December 2013. The index date was defined as the date of CHB diagnosis. The exclusion criteria were as follows: a history of CHB on the index date or liver cancer prior to or within 2 years of the index date (n = 316 073), death within 2 years of the index date (n = 13 723), <19 or >80 years of age on the index date (n = 17 875), and missing demographic data (n = 8639). A total of 414 074 patients was included in the study (Figure 1).

Cirrhosis was clinically defined as having a disease code for cirrhosis or clinical features of portal hypertension such as ascites or varices. Among the patients with a cirrhosis disease code, those with at least two or more visits to an outpatient clinic or one admission were considered to have cirrhosis. Patients prescribed nonselective beta‐blockers or spironolactone for >1 month or who received esophageal band ligation or paracentesis were also considered to have cirrhosis.

This study was approved by the Institutional Review Board of Kyung Hee University, Seoul, ROK, and was performed in accordance with the ethical guidelines of the 1975 Declaration of Helsinki.

### 2.3 Definition of regular follow‐up

We counted the number of visits to a hospital or other medical facility within 2 years of the index date. One year comprises four quarters, thus follow‐up visits were counted over eight quarters (Q1‐Q8). Several visits in the same quarter were considered one visit and only visits related to CHB (B18.0 or B18.1) were counted. The number of visits in the eight quarters were summed. Patients were categorized into the following three follow‐up groups: Regular follow‐up, more than four visits; irregular follow‐up, one to three visits; and no follow‐up, zero visits.

### 2.4 Primary and secondary outcomes

The primary outcome was the rate of liver cancer mortality. The secondary outcomes were the incidence of liver cancer and all‐cause mortality rate. The association between clinical outcomes and regular follow‐up was investigated. The association between the number of follow‐up visits and clinical outcomes was also investigated. Follow‐up continued until the date of death or the end of the study period (December 31, 2017).

Liver cancer was defined as a disease code of C22.0, C22.1, or C22.9 in the NHIS database. We included intrahepatic cholangiocarcinoma (C22.1) in the analysis because CHB is also associated with it.^19^, ^20^ Information on date of death was available, but not on cause of death in the cohort. So, liver cancer mortality was defined as death with a history of hospital admission due to liver cancer within 3 months from the time of death.

### 2.5 Statistical analyses

Differences in categorical variables between groups were compared using the chi‐squared test. Continuous variables were compared using the unpaired two‐tailed *t* test. The rate of liver cancer mortality, incidence of liver cancer, and the all‐cause mortality rate were estimated using the Kaplan‐Meier method and were compared using the log‐rank test.

A Cox proportional hazard regression analysis was performed to assess the risks of primary and secondary outcomes associated with regular follow‐up. Crude and adjusted hazard ratios (HRs) with 95% confidence intervals (CIs) are provided. The multivariable analysis included the following variables: gender, age, income (medical aid as the lowest income level; quintiles 1‐5), disability (yes vs no), residence area (urban = Seoul, Incheon, or Gyeonggi‐do vs rural = other areas), medical facility (hospital = ≥30 beds, clinic = <30 beds, others), and Charlson comorbidity index (CCI; 0, 1, 2, or more).^21^

Statistical analysis was performed using SAS software (ver. 9.4; SAS Institute, Cary, NC, USA). Two‐sided *P*‐values of <.05 were considered to indicate statistical significance.

## 3 RESULTS

### 3.1 Baseline characteristics of the patients

A total of 414 074 adult patients with CHB was enrolled in this study. The mean age of the patients was 48.0 years, and 53.4% were males. Most of the patients were in their 50s (26.6%) or 40s (25.8%). The proportion of patients receiving medical aid was 4.7%. The vast majority of the patients was not disabled (93.0%). About half of the patients resided in an urban area (44.6%) and were diagnosed at a hospital (51.0%). The CCI, based on the number of comorbidities in the year before the index date, was 0 for 54.5% of the patients, 1 for 26.2% of patients, and ≥2 for 19.3% of the patients (Table 1).

**Table 1:** Baseline characteristics of the patients

	Total	Patients with cirrhosis	Patients without cirrhosis	*P*
Number of patients	414 074	26 086	387 988	
Gender				
Men	221 032 (53.4)	17 212 (66.0)	203 820 (52.5)	<0.001
Women	193 042 (46.6)	8874 (34.0)	184 168 (47.5)	
Mean age, years (SD)	48.0 (13.2)	53.9 (10.1)	47.6 (13.2)	<0.001
Age group				
20‐29	34 369 (8.3)	191 (0.7)	34 178 (8.8)	<0.001
30‐39	81 728 (19.7)	1661 (6.4)	80 067 (20.6)	
40‐49	106 888 (25.8)	6846 (26.3)	100 042 (25.8)	
50‐59	110 065 (26.6)	10 175 (39.0)	99 890 (25.8)	
60‐69	54 548 (13.2)	5172 (19.8)	49 376 (12.7)	
≥70	26 476 (6.4)	2041 (7.8)	24 435 (6.3)	
Income^a^				
Medical aid	19 607 (4.7)	1769 (6.8)	17 838 (4.6)	<0.001
Quintile 1	58 237 (14.1)	3795 (14.5)	54 442 (14.0)	
Quintile 2	65 346 (15.8)	4003 (15.3)	61 343 (15.8)	
Quintile 3	77 461 (18.7)	4604 (17.7)	72 857 (18.8)	
Quintile 4	89 944 (21.7)	5271 (20.2)	84 673 (21.8)	
Quintile 5	103 479 (25.0)	6644 (25.5)	96 835 (25.0)	
Disabled person				
No	385 000 (93.0)	23 556 (90.3)	361 444 (93.2)	<0.001
Yes	29 074 (7.0)	2530 (9.7)	26 544 (6.8)	
Residence area				
Urban	184 628 (44.6)	12 503 (47.9)	172 125 (44.4)	<0.001
Rural	229 446 (55.4)	13 583 (52.1)	215 863 (55.6)	
Medical facility				
Hospitals^b^	211 151 (51.0)	19 671 (75.4)	191 480 (49.4)	<0.001
Private clinics	202 374 (48.9)	6390 (24.5)	195 984 (50.5)	
Others	549 (0.1)	25 (0.1)	524 (0.1)	
CCI				
0	225 801 (54.5)	11 529 (44.2)	214 272 (55.2)	<0.001
1	108 251 (26.2)	7168 (27.5)	101 083 (26.1)	
≥2	80 022 (19.3)	7389 (28.3)	72 633 (18.7)	

Note

Data are presented as numbers with percentages in parentheses unless otherwise indicated.

Abbreviations: CCI, Charlson comorbidity index; SD, standard deviation.

^a^ Income ranked by insurance premium. Subjects under medical aid pay no premium, which indicated the lowest income level.

^b^ Hospitals include general or specialist hospitals with > 30 beds.

Of the patients, 6.3% (n = 26 086) had cirrhosis. The patients with cirrhosis were older (53.9 vs 47.6 years; *P* < .001), predominantly male (66.0% vs 52.5%; *P* < .001), and had a higher frequency of disability (9.7% vs 6.8%; *P* < .001) compared to those without cirrhosis (Table 1).

### 3.2 Follow‐up groups

Of the patients, 22.9%, 58.0%, and 19.1% were in the regular, irregular, and no follow‐up groups respectively (Table 2). Patients were more likely to have irregular or no follow‐up when they were female, >60 years of age, of low socioeconomic status, disabled, lived in a rural area, had a high comorbidity rate, or did not have cirrhosis (Table 2). The distribution of patients according to number of visits is shown in Table S1.

**Table 2:** Clinical characteristics of the patients according to follow‐up pattern

	No FU	Irregular FU	Regular FU	*P*
Number of patients	79 333 (19.1)	239 960 (58.0)	94 781 (22.9)	
Gender				
Men	40 502 (18.3)	123 865 (56.1)	56 665 (25.6)	<0.001
Women	38 831 (20.1)	116 095 (60.1)	38 116 (19.8)	
Mean age, years (SD)	51.0 (14.0)	46.8 (13.0)	48.5 (11.7)	<0.001
Age group				
20‐29	5259 (15.3)	23 761 (69.1)	5349 (15.6)	<0.001
30‐39	12 803 (15.7)	51 750 (63.3)	17 175 (21.0)	
40‐49	18 139 (17.0)	62 232 (58.2)	26 517 (24.8)	
50‐59	20 677 (18.8)	60 424 (54.9)	28 964 (26.3)	
60‐69	13 039 (23.9)	29 066 (53.3)	12 443 (22.8)	
≥70	9416 (35.5)	12 727 (48.1)	4333 (16.4)	
Income^a^				
Medical aid	5332 (27.2)	10 350 (52.8)	3925 (20.0)	<0.001
Quintile 1	11 827 (20.3)	33 831 (58.1)	12 579 (21.6)	
Quintile 2	12 684 (19.4)	38 140 (58.4)	14 522 (22.2)	
Quintile 3	14 541 (18.8)	45 133 (58.2)	17 787 (23.0)	
Quintile 4	16 755 (18.6)	52 173 (58.0)	21 016 (23.4)	
Quintile 5	18 194 (17.6)	60 333 (58.3)	24 952 (24.1)	
Disabled person				
No	71 061 (18.5)	225 325 (58.5)	88 614 (23.0)	<0.001
Yes	8272 (28.5)	14 635 (50.3)	6167 (21.2)	
Residence area				
Urban	28 876 (15.6)	110 429 (59.8)	45 323 (24.6)	<0.001
Rural	50 457 (22.0)	129 531 (56.4)	49 458 (21.6)	
Medical facility				
Hospitals^b^	57 367 (27.2)	99 521 (47.1)	54 263 (25.7)	<0.001
Private clinics	21 878 (10.8)	140 096 (69.2)	40 400 (20.0)	
Others	88 (16.0)	343 (62.5)	118 (21.5)	
CCI				
0	38 963 (17.2)	138 367 (61.3)	48 471 (21.5)	<0.001
1	20 035 (18.5)	61 547 (56.9)	26 669 (24.6)	
≥2	20 335 (25.4)	40 046 (50.0)	19 641 (24.6)	
Cirrhosis				
Yes	2256 (8.6)	7944 (30.5)	15 886 (60.9)	<0.001
No	77 077 (19.9)	232 016 (59.8)	78 895 (20.3)	

Note

Data are presented as numbers with percentages in parentheses unless otherwise indicated.

Abbreviations: CCI, Charlson comorbidity index; FU, follow‐up; SD, standard deviation.

^a^ Income ranked by insurance premium. Subjects under medical aid pay no premium, which indicated the lowest income level.

^b^ Hospitals include general or specialist hospitals with > 30 beds.

### 3.3 Regular follow‐up and liver cancer mortality

**Figure 2:**
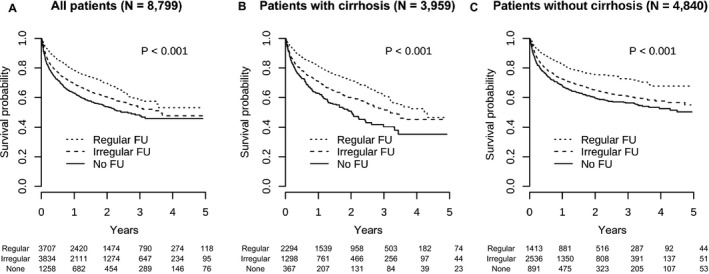
Kaplan‐Meier survival curves. The proportion free of death due to liver cancer of all patients (A), patients with cirrhosis (B), and patients without cirrhosis (C)

During the mean follow‐up duration of 6.7 years, liver cancer was diagnosed in 8799 patients (2.1%). Of them, 2555 patients (29.0%) died from liver cancer. Of the 3707 and 1258 patients in the regular and no follow‐up groups, 856 (23.1%) and 488 (38.8%) died due to liver cancer respectively (*P* < .001; Figure 2A). The survival benefit of regular follow‐up was consistent in patients with and without cirrhosis (*P* < .001; Figure 2B and C). In multivariable analysis with adjustment for age, gender, income, disability, residence area, type of medical facility, and CCI, regular follow‐up was independently associated with decreased risk of liver cancer mortality (HR, 0.56; 95% CI, 0.50‐0.63; *P* < .001; Table 3). This beneficial effect of regular follow‐up was consistent in patients with (HR, 0.56; 95% CI, 0.47‐0.67; *P* < .001) and without (HR, 0.56; 95% CI, 0.48‐0.66; *P* < .001) cirrhosis.

**Table 3:** Association between liver cancer mortality and regular follow‐up in patients with liver cancer

FU	Liver cancer mortality			
	No. of patients	No. of events	Crude	Adjusted model^a^
			HR (95% CI)	HR (95% CI)
All patients (n = 8799)				
No FU	1258	488	Reference	Reference
Irregular FU	3834	1211	0.84 (0.76‐0.94)	0.87 (0.78‐0.97)
Regular FU	3707	856	0.55 (0.49‐0.62)	0.56 (0.50‐0.63)
Patients with cirrhosis (n = 3959)				
No FU	367	160	Reference	Reference
Irregular FU	1298	456	0.83 (0.69‐0.99)	0.87 (0.72‐1.05)
Regular FU	2294	562	0.53 (0.44‐0.63)	0.56 (0.47‐0.67)
Patients without cirrhosis (n = 4840)				
No FU	891	328	Reference	Reference
Irregular FU	2536	755	0.83 (0.73‐0.94)	0.85 (0.74‐0.97)
Regular FU	1413	294	0.53 (0.45‐0.62)	0.56 (0.48‐0.66)

Abbreviations: CI, confidence interval; FU, follow‐up; HR, hazard ratio.

^a^ A Cox proportional hazards model was used to adjust for age, gender, income, disability, residence area, hospital type, and CCI.

Mortality risk was evaluated according to the number of follow‐up visits during the 2 years following the index date, and seven visits was associated with the lowest risk of death due to liver cancer in the multivariable analysis (HR, 0.47; 95% CI, 0.38‐0.57, *P* < .001; Tables S2 and S3). Over a 2‐year period, seven visits would occur over approximately 3‐month intervals, whereas eight visits imply a visit at least every 3 months or more frequently. Patients with more severe disease, who require more frequent follow‐ups, might be included in this subgroup. In patients with cirrhosis, those who underwent seven follow‐up visits showed the lowest risk of mortality. In patients without cirrhosis, roughly 3‐ to 6‐month intervals were associated with a lower risk of liver cancer mortality (Table S3).

### 3.4 Regular follow‐up and incidence of liver cancer

During the study period, the annual incidence of liver cancer was 3.19 per 1000 person‐years (PY). In a multivariable analysis, regular follow‐up was associated with an increased incidence of liver cancer (HR, 1.54; 95% CI, 1.44‐1.65, *P* < .001; Table 4).

**Table 4:** Association between incidence of liver cancer and regular follow‐up

FU	Incidence of liver cancer			
	No. of patients	No. of events	Crude	Adjusted model^a^
			HR (95% CI)	HR (95% CI)
All patients (n = 414 074)				
No FU	79 333	1258	Reference	Reference
Irregular FU	239 960	3834	1.10 (1.03‐1.17)	1.29 (1.21‐1.38)
Regular FU	94 781	3707	2.89 (2.71‐3.08)	1.54 (1.44‐1.65)
Patients with cirrhosis (n = 26 086)				
No FU	2256	367	Reference	Reference
Irregular FU	7944	1298	0.95 (0.84‐1.06)	1.07 (0.95‐1.20)
Regular FU	15 886	2294	0.92 (0.82‐1.03)	1.06 (0.95‐1.19)
Patients without cirrhosis (n = 387 988)				
No FU	77 077	891	Reference	Reference
Irregular FU	232 016	2536	1.04 (0.96‐1.12)	1.38 (1.27‐1.49)
Regular FU	78 895	1413	1.80 (1.65‐1.95)	1.95 (1.79‐2.13)

Abbreviations: CI, confidence interval; FU, follow‐up; HR, hazard ratio.

^a^ A Cox proportional hazards model was used to adjust for age, sex, income, disability, residence area, hospital type, and CCI.

Of the patients with cirrhosis, 3959 (15.2%) developed liver cancer; ie, an incidence of 24.99 per 1000 PY. The incidences were 23.79, 26.61, and 27.82 per 1000 PY in the regular, irregular, and no follow‐up groups respectively. In multivariable analysis, regular follow‐up was not associated with an increased incidence of liver cancer for the patients with cirrhosis (HR, 1.06; 95% CI, 0.95‐1.19, *P* = .31; Table 4).

Of patients without cirrhosis, 4840 (1.2%) developed liver cancer; ie, an incidence of 1.86 per 1000 PY. The incidences were 2.72, 1.63, and 1.70 per 1000 PY in the regular, irregular, and no follow‐up groups respectively. In multivariable analysis, regular follow‐up was associated with an increased incidence of liver cancer for the patients without cirrhosis (HR, 1.95; 95% CI, 1.79‐2.13; *P* < .001; Table 4).

The incidence of liver cancer increased with increasing number of follow‐up visits only for the patients without cirrhosis (Table S4).

### 3.5 Regular follow‐up and all‐cause mortality

In total, 15 391 (3.7%) patients died during the study period; the annual all‐cause mortality rate was 5.55 per 1000 PY. The mortality rates were 5.40, 4.20, and 9.78 per 1000 PY for patients with regular, irregular, and no follow‐up respectively. In multivariable analysis, regular follow‐up was independently associated with a decreased risk of all‐cause mortality (HR, 0.60; 95% CI, 0.57‐0.63, *P* < .001; Table 5). This benefit of regular follow‐up was consistent between the patients with (HR, 0.42; 95% CI, 0.38‐0.46, *P* < .001) and without (HR, 0.70; 95% CI, 0.66‐0.74, *P* < .001; Table 5) cirrhosis.

**Table 5:** Association between all‐cause mortality and regular follow‐up

FU	All‐cause mortality			
	No. of patients	No. of events	Crude	Adjusted model^a^
			HR (95% CI)	HR (95% CI)
All patients (n = 414 074)				
No FU	79 333	5271	Reference	Reference
Irregular FU	239 960	6759	0.43 (0.42‐0.45)	0.74 (0.71‐0.76)
Regular FU	94 781	3361	0.56 (0.54‐0.59)	0.60 (0.57‐0.63)
Patients with cirrhosis (n = 26 086)				
No FU	2256	690	Reference	Reference
Irregular FU	7944	1378	0.53 (0.48‐0.58)	0.71 (0.65‐0.78)
Regular FU	15 886	1471	0.29 (0.26‐0.32)	0.42 (0.38‐0.46)
Patients without cirrhosis (n = 387 988)				
No FU	77 077	4581	Reference	Reference
Irregular FU	232 016	5381	0.40 (0.38‐0.42)	0.75 (0.72‐0.78)
Regular FU	78 895	1890	0.42 (0.40‐0.45)	0.70 (0.66‐0.74)

Abbreviations: CI, confidence interval; FU, follow‐up; HR, hazard ratio.

^a^ A Cox proportional hazards model was used to adjust for age, gender, income, disability, residence area, hospital type, and CCI.

The number of follow‐up visits was negatively associated with all‐cause mortality. Eight follow‐up visits, indicating that the patient visited a medical facility every 3 months, was associated with the lowest risk of all‐cause mortality (Table S5).

### 3.6 Regular follow‐up and curative treatment

The insurance claim codes for curative treatments were investigated (Table S6). Of the 8799 patients, 1640 (18.6%) received curative treatments such as hepatic resection, liver transplantation, or radiofrequency ablation. Compared with patients in the irregular and no follow‐up groups, patients in the regular follow‐up group received a higher rate of curative treatment irrespective of the presence of cirrhosis (regular vs irregular: 23.1% vs 15.5%, *P* < .001; regular vs no follow‐up; 23.1% vs 15.1%; *P* < .001; Table 6). There was no significant difference between the irregular and no follow‐up groups.

**Table 6:** Proportion of patients on curative treatment in each follow‐up group

FU	Curative treatment	Other treatments	*P* ^a^	*P* ^b^	*P* ^c^
All patients (n = 8799)					
No FU	190 (15.1)	1068 (84.9)	0.72	<0.001	<0.001
Irregular FU	595 (15.5)	3239 (84.5)			
Regular FU	855 (23.1)	2852 (76.9)			
Patients with cirrhosis (n = 3959)					
No FU	63 (17.2)	304 (82.8)	0.40	<0.001	0.001
Irregular FU	248 (19.1)	1050 (80.9)			
Regular FU	582 (25.4)	1712 (74.6)			
Patients without cirrhosis (n = 4840)					
No FU	127 (14.2)	764 (85.8)	0.67	<0.001	0.002
Irregular FU	347 (13.7)	2189 (86.3)			
Regular FU	273 (19.3)	1140 (80.7)			

Note

Data are presented as numbers with percentages in parentheses unless otherwise indicated.

^a^ *P*‐value for comparison between the no follow‐up and irregular follow‐up groups.

^b^ *P*‐value for comparison between the irregular follow‐up and regular follow‐up groups.

^c^ *P*‐value for comparison between the no follow‐up and regular follow‐up groups.

## 4 DISCUSSION

This study is the first to confirm the beneficial effect of regular clinic follow‐up on liver cancer mortality as well as all‐cause mortality in patients with CHB using a large population‐based observational study. Patients with regular follow‐up had a 44% lower risk of death due to liver cancer compared with those who had no follow‐up. This is strong evidence in support of recommending regular follow‐up regardless of whether the patient exhibits symptoms. In clinical practice, there are many barriers to regular follow‐up such as scheduling process, costs, or transportation difficulties.^22^ Many patients miss scheduled visits because of low motivation due to the asymptomatic nature of the disease and lack of client education on the impact that follow‐up can have on their health. The demonstrated benefit of regular follow‐up in this study may motivate these patients to maintain their follow‐up schedule and serve as strong evidence for health care providers to educate their patients. Given the widespread nonadherence to regular follow‐up, our findings highlight that novel and effective strategies are needed in most patients with CHB.

Low adherence to regular follow‐up can delay medical interventions and worsen the prognosis of patients with CHB. The study cohort had a very low (23%) rate of regular follow‐up. Because > 99% of the population of the ROK is covered by the NHIS and access to medical care is guaranteed, this low rate of regular follow‐up is alarming.^23^, ^24^ Patients who were female, >60 years of age, of low socioeconomic status, disabled, lived in a rural area, had a high comorbidity rate, or did not have cirrhosis were more likely to miss regular follow‐up. Some of these factors are established predictors of inadequate surveillance.^10^, ^13^, ^25^, ^26^ Of note, cirrhosis was strongly associated with compliance with regular follow‐up, possibly due to awareness of the severity of the disease.^10^, ^25^ The reported underutilization of HCC surveillance together with our findings highlight the need for strategies to overcome these barriers and promote regular follow‐up.^14^, ^27^, ^28^

We found that the optimal number of follow‐up visits associated with the lowest risk of liver cancer mortality was seven, conducted at ~3‐month intervals over a 2‐year period, after the diagnosis of CHB. Whereas the benefit of seven follow‐up visits was obvious in patients with cirrhosis, this effect was less remarkable in patients without cirrhosis. Taken together, follow‐up at 3‐month intervals for patients with cirrhosis and 3‐ to 6‐month intervals for patients without cirrhosis would be optimal. Yet, future studies on the optimal number of follow‐up visits are needed. The slightly increased rate of liver cancer mortality in patients who made more than eight visits may have derived from the severity of their liver disease requiring short‐term follow‐up.

Regular follow‐up was associated with an increased rate of curative treatment and incidence of liver cancer. Regular follow‐up may have increased the tumor‐detection rate by reducing the surveillance interval, and early detection of tumors may have increased the likelihood of receiving curative treatment.^29^ As reported earlier, a greater number of hospital visits is reportedly linked to improved surveillance.^30^ Taken together, the increased incidence of liver cancer and the decreased rate of liver cancer mortality in the regular follow‐up group imply that better surveillance assisted by regular follow‐up improved patient prognosis.

This study has several limitations. First, its observational design may have resulted in unmeasured confounders for the analysis. Yet, the population‐based historical cohort enabled investigation of the impact of follow‐up on the long‐term prognosis at all levels of the healthcare system. Because the study was not limited to a single center, we could assess the follow‐up patterns of the general population using the NHIS database, which covers >99% of the population of the ROK. Second, the possibility that some patients with cirrhosis were included in the noncirrhosis group cannot be ruled out. Because the clinical and radiological data used to diagnose cirrhosis were not available, a rather strict approach was adopted. In prior studies ~80%‐90% of the patients with liver cancer also had cirrhosis, compared to 45% in this study.^31^, ^32^ However, the effect of regular follow‐up on prognosis was consistent in patients with and without cirrhosis. Third, this study was not able to investigate whether any antiviral therapy or surveillance testing attributed to the reduced liver cancer mortality or not since the given dataset was void of such details.^33^, ^34^ Sensitive personal information and prescription details were not available when the analysis was performed. In addition, we could not directly investigate the association between regular follow‐up and compliance of surveillance testing for HCC in the cohort. However, according to a recent study showing strong association between surveillance rate and medical visits, we can consider this assumption as acceptable with some confidence.^30^ Optimal surveillance for HCC was associated with earlier detection, more curative treatment, and better survival after adjusting lead‐time bias in a prospective study.^29^ As an alternative to surveillance testing, the frequency of curative treatment was investigated. The information on the divisions or physicians patients visited matters, however, this study was not able to include them since such information was not available in the given dataset. Fourth, we defined liver cancer mortality only in patients who had admitted to hospital before 3 months of death since the cohort did not have any data on cause of death. This strict definition might have been associated with relatively low liver cancer mortality (29%). Yet, the impact of regular follow‐up was consistently observed for both liver cancer mortality and overall mortality. In addition, the study excluded the deaths occurred within 2 years from the index date since the aim was to investigate the association between the follow‐up pattern and liver cancer mortality. These deaths may have been included late presentation of HCC or cholangiocarcinoma. Fifth, we could not explain the higher incidence of liver cancer in the regular follow‐up group without cirrhosis. Surveillance testing might detect more slowly growing tumors. The group may have included more patients with severe liver disease such as requiring antiviral therapy. Undiagnosed cirrhosis may have affected the outcome for that group. Finally, we analyzed only Koreans with CHB, most of whom are infected with genotype C HBV by vertical transmission.^35^ Genotype C HBV has a higher risk of disease progression than other genotypes.^36^, ^37^ This limits the ability to generalize these findings.

In conclusion, regular follow‐up reduces the risk of liver cancer mortality as well as all‐cause mortality in patients with CHB. Also, being female, age <40 or >60 years, low socioeconomic status, being disabled, living in a rural area, a high comorbidity rate, and the absence of cirrhosis were barriers to regular follow‐up. The findings provide a solid foundation for promoting greater adherence to medical follow‐up by educating CHB patients on the importance of regular follow‐up. Given its benefit, strategies to promote regular follow‐up should be developed and implemented.

## CONFLICTS OF INTEREST

The authors have nothing to disclose relevant to this manuscript.

## AUTHORS’ CONTRIBUTIONS

All of the authors have full access to the data used in the study and take responsibility for the integrity of the data and the accuracy of data analysis. All authors were responsible for the design of the study; acquisition, analysis, and interpretation of the data; and drafting of the manuscript. JJ Shim, GA Kim, and IH Oh contributed to data analysis and interpretation, and drafting of the manuscript, and CH Oh, JW Kim, J M, and BH Kim critically revised important intellectual content of the manuscript. All authors approved submission of the final version.

## Supporting information

Table S1‐S6

## Data Availability

Data sharing is not applicable to this article due to technical or time limitations.
