# Supplementary material for: Reduced liver cancer mortality with regular clinic follow‐up among patients with chronic hepatitis B: A nationwide cohort study
Source: Cancer Med. 2020 Aug 28;9(20):7781–91. doi: 10.1002/cam4.3421 (PMC7571840; doi:10.1002/cam4.3421)
Supplement: Supplementary file 1 — Table S1‐S6 [file CAM4-9-7781-s001.docx]

**Supporting Table 1. Number of follow‑up visits.**

| **FU pattern** | **Visit**  **number** | **All patients**  **(n = 414,074)** | **Patients with cirrhosis**  **(n = 26,086)** | **Patients without cirrhosis**  **(n = 387,988)** |
| --- | --- | --- | --- | --- |
| No FU | 0 | 79,333 (19.2) | 2,256 (8.6) | 77,077 (19.9) |
| Irregular FU | 1 | 152,021 (36.7) | 3,399 (13.0) | 148,622 (38.3) |
|  | 2 | 57,624 (13.9) | 2,430 (9.3) | 55,194 (14.2) |
|  | 3 | 30,315 (7.3) | 2,115 (8.1) | 28,200 (7.3) |
| Regular FU | 4 | 20,495 (4.9) | 2,090 (8.0) | 18,405 (4.7) |
|  | 5 | 15,504 (3.7) | 2,174 (8.3) | 13,330 (3.4) |
|  | 6 | 13,785 (3.3) | 2,494 (9.6) | 11,291 (2.9) |
|  | 7 | 14,848 (3.6) | 3,105 (11.9) | 11,743 (3.0) |
|  | 8 | 30,149 (7.3) | 6,023 (23.1) | 24,126 (6.2) |

All visits in the same quarter were considered one visit; only visits related to chronic hepatitis B (B180 or B181) were considered. Data are presented as numbers with percentages in parentheses.

Abbreviations: FU, follow-up

**Supporting Table 2. Rates of liver cancer mortality according to number of follow‑up visits in patients with liver cancer.**

| **Visit number** | **All (n = 8,799)** | **Patients with cirrhosis (n = 3,959)** | **Patients without cirrhosis**  **(n = 4,840)** |
| --- | --- | --- | --- |
|  | **No. of deaths / no. of patients (%)** | **No. of deaths / no. of patients (%)** | **No. of deaths / no. of patients (%)** |
| 0 | 488 / 1,258 (38.8) | 160 / 367 (43.6) | 328 / 891 (36.8) |
| 1 | 680 / 2,072 (32.8) | 213 / 602 (35.4) | 467 / 1,470 (31.8) |
| 2 | 342 / 1,074 (31.8) | 143 / 386 (37.1) | 199 / 688 (28.9) |
| 3 | 189 / 688 (27.5) | 100 / 310 (32.3) | 89 / 378 (23.5) |
| 4 | 120 / 504 (23.8) | 70 / 276 (25.4) | 50 / 228 (21.9) |
| 5 | 128 / 520 (24.6) | 80 / 288 (27.8) | 48 / 232 (20.7) |
| 6 | 127 / 536 (23.7) | 78 / 313 (24.9) | 49 / 223 (22.0) |
| 7 | 134 / 664 (20.2) | 92 / 453 (20.3) | 42 / 211 (19.9) |
| 8 | 347 / 1,483 (23.4) | 242 / 964 (25.1) | 105 / 519 (20.2) |

**Supporting Table 3. Risk of liver cancer mortality according to number of follow‑up visits.**

| **Method** | **Visit number** | **All (n = 8,799)** | **Patients with cirrhosis**  **(n = 3,959)** | **Patients without cirrhosis (n = 4,840)** |
| --- | --- | --- | --- | --- |
|  |  | **HR (95% CI)** | **HR (95% CI)** | **HR (95% CI)** |
| **Crude** | 0 | Reference | Reference | Reference |
|  | 1 | 0.90 (0.80–1.01) | 0.86 (0.70–1.06) | 0.90 (0.78–1.03) |
|  | 2 | 0.86 (0.74–0.98) | 0.87 (0.69–1.09) | 0.81 (0.68–0.97) |
|  | 3 | 0.68 (0.57–0.80) | 0.72 (0.56–0.93) | 0.60 (0.48–0.76) |
|  | 4 | 0.58 (0.48–0.71) | 0.55 (0.42–0.73) | 0.56 (0.42–0.76) |
|  | 5 | 0.59 (0.48–0.71) | 0.60 (0.46–0.79) | 0.51 (0.37–0.69) |
|  | 6 | 0.59 (0.49–0.72) | 0.57 (0.43–0.75) | 0.57 (0.42–0.76) |
|  | 7 | 0.46 (0.38–0.56) | 0.41 (0.32–0.53) | 0.50 (0.36–0.69) |
|  | 8 | 0.56 (0.49–0.64) | 0.54 (0.44–0.66) | 0.51 (0.41–0.64) |
| **Adjusted model^a^** | 0 | Reference | Reference | Reference |
|  | 1 | 0.93 (0.82–1.05) | 0.91 (0.74–1.12) | 0.92 (0.79–1.06) |
|  | 2 | 0.88 (0.76–1.01) | 0.90 (0.72–1.13) | 0.84 (0.70–1.00) |
|  | 3 | 0.70 (0.59–0.82) | 0.77 (0.60–0.99) | 0.63 (0.49–0.79) |
|  | 4 | 0.60 (0.49–0.73) | 0.59 (0.45–0.79) | 0.60 (0.45–0.81) |
|  | 5 | 0.60 (0.50–0.73) | 0.65 (0.50–0.86) | 0.53 (0.39–0.72) |
|  | 6 | 0.60 (0.49–0.73) | 0.61 (0.47–0.81) | 0.59 (0.44–0.80) |
|  | 7 | 0.47 (0.38–0.57) | 0.45 (0.34–0.58) | 0.52 (0.38–0.72) |
|  | 8 | 0.56 (0.49–0.65) | 0.57 (0.46–0.70) | 0.55 (0.44–0.69) |

^a^A Cox proportional hazards model was used to adjust for age, sex, income, disability, residence area, hospital type, and Charlson comorbidity index (CCI).

Abbreviations: CI, confidence interval; HR, hazard ratio

**Supporting Table 4. Risk of liver cancer incidence according to number of follow‑up visits.**

| **Method** | **Visit number** | **All (n = 414,074)** | **Patients with cirrhosis (n = 26,086)** | **Patients without cirrhosis (n = 387,988)** |
| --- | --- | --- | --- | --- |
|  |  | **HR (95% CI)** | **HR (95% CI)** | **HR (95% CI)** |
| **Crude** | 0 | Reference | Reference | Reference |
|  | 1 | 0.94 (0.87–1.00) | 1.06 (0.93–1.21) | 0.94 (0.86–1.02) |
|  | 2 | 1.29 (1.19–1.40) | 0.89 (0.77–1.03) | 1.19 (1.08–1.32) |
|  | 3 | 1.57 (1.44–1.73) | 0.84 (0.72–0.98) | 1.28 (1.14–1.45) |
|  | 4 | 1.76 (1.59–1.95) | 0.76 (0.65–0.89) | 1.22 (1.06–1.42) |
|  | 5 | 2.44 (2.20–2.70) | 0.82 (0.70–0.96) | 1.73 (1.50–2.00) |
|  | 6 | 2.84 (2.57–3.15) | 0.79 (0.68–0.91) | 1.96 (1.69–2.27) |
|  | 7 | 3.33 (3.03–3.66) | 0.94 (0.82–1.08) | 1.80 (1.55–2.09) |
|  | 8 | 3.75 (3.48–4.04) | 1.07 (0.95–1.21) | 2.20 (1.98–2.46) |
| **Adjusted model^a^** | 0 | Reference | Reference | Reference |
|  | 1 | 1.24 (1.15–1.33) | 1.18 (1.03–1.34) | 1.28 (1.17–1.39) |
|  | 2 | 1.37 (1.26–1.48) | 0.99 (0.86–1.14) | 1.54 (1.39–1.70) |
|  | 3 | 1.37 (1.25–1.51) | 0.97 (0.84–1.13) | 1.56 (1.39–1.77) |
|  | 4 | 1.23 (1.11–1.37) | 0.88 (0.76–1.03) | 1.39 (1.20–1.61) |
|  | 5 | 1.46 (1.31–1.62) | 0.95 (0.81–1.11) | 1.94 (1.68–2.24) |
|  | 6 | 1.49 (1.34–1.65) | 0.94 (0.81–1.10) | 2.12 (1.83–2.46) |
|  | 7 | 1.55 (1.41–1.71) | 1.09 (0.95–1.25) | 1.92 (1.65–2.23) |
|  | 8 | 1.78 (1.65–1.93) | 1.20 (1.07–1.36) | 2.31 (2.07–2.58) |

^a^A Cox proportional hazards model was used to adjust for age, sex, income, disability, residence area, hospital type, and CCI.

**Supporting Table 5. Risk of all-cause mortality according to number of follow‑up visits.**

| **Method** | **Visit number** | **All (n = 414,074)** | **Patients with cirrhosis (n = 26,086)** | **Patients without cirrhosis**  **(n = 387,988)** |
| --- | --- | --- | --- | --- |
|  |  | **HR (95% CI)** | **HR (95% CI)** | **HR (95% CI)** |
| **Crude** | **0** | Reference | Reference | Reference |
|  | **1** | 0.44 (0.42–0.45) | 0.66 (0.60–0.74) | 0.42 (0.40–0.43) |
|  | **2** | 0.42 (0.40–0.44) | 0.46 (0.40–0.52) | 0.38 (0.35–0.40) |
|  | **3** | 0.44 (0.41–0.47) | 0.41 (0.36–0.47) | 0.35 (0.33–0.39) |
|  | **4** | 0.50 (0.46–0.54) | 0.34 (0.29–0.39) | 0.40 (0.36–0.44) |
|  | **5** | 0.57 (0.52–0.62) | 0.32 (0.27–0.37) | 0.44 (0.39–0.49) |
|  | **6** | 0.58 (0.53–0.63) | 0.28 (0.24–0.33) | 0.43 (0.38–0.49) |
|  | **7** | 0.61 (0.56–0.67) | 0.26 (0.23–0.30) | 0.47 (0.42–0.53) |
|  | **8** | 0.57 (0.53–0.61) | 0.28 (0.25–0.31) | 0.40 (0.37–0.44) |
| **Adjusted model^a^** | **0** | Reference | Reference | Reference |
|  | **1** | 0.79 (0.75–0.82) | 0.85 (0.76–0.94) | 0.79 (0.76–0.83) |
|  | **2** | 0.68 (0.64–0.72) | 0.61 (0.54–0.70) | 0.70 (0.66–0.75) |
|  | **3** | 0.63 (0.59–0.68) | 0.58 (0.51–0.67) | 0.63 (0.58–0.69) |
|  | **4** | 0.61 (0.56–0.67) | 0.49 (0.42–0.56) | 0.65 (0.59–0.72) |
|  | **5** | 0.64 (0.58–0.70) | 0.46 (0.40–0.54) | 0.74 (0.66–0.82) |
|  | **6** | 0.59 (0.54–0.65) | 0.42 (0.36–0.49) | 0.70 (0.62–0.79) |
|  | **7** | 0.58 (0.53–0.63) | 0.38 (0.33–0.44) | 0.77 (0.69–0.87) |
|  | **8** | 0.57 (0.53–0.61) | 0.40 (0.36–0.45) | 0.68 (0.62–0.74) |

^a^A Cox proportional hazards model was used to adjust for age, sex, income, disability, residence area, hospital type, and CCI.

**Supporting Table 6. Insurance claim codes of curative treatments.**

| **Treatment** | **Claim codes** |
| --- | --- |
| Hepatic resection | Q7221, Q7222, Q7223, Q7224 |
| Liver transplantation | Q8040, Q8041, Q8042, Q8043, Q8044, Q8045, Q8046, Q8047, Q8048, Q8049, Q8050 |
| Radiofrequency ablation | Q7280, Q7281, QZ841 |
